# Comparative epidemiology of gestational diabetes in ethnic Chinese from Shanghai birth cohort and growing up in Singapore towards healthy outcomes cohort

**DOI:** 10.1186/s12884-021-04036-5

**Published:** 2021-08-18

**Authors:** Evelyn Xiu Ling Loo, Yuqing Zhang, Qai Ven Yap, Guoqi Yu, Shu E Soh, See Ling Loy, Hui Xing Lau, Shiao-Yng Chan, Lynette Pei-Chi Shek, Zhong-Cheng Luo, Fabian Kok Peng Yap, Kok Hian Tan, Yap Seng Chong, Jun Zhang, Johan Gunnar Eriksson

**Affiliations:** 1 Singapore Institute for Clinical Sciences (SICS), Agency for Science, Technology and Research (A*STAR), 30 Medical Drive, Singapore, 117609 Singapore; 2 Department of Paediatrics, Yong Loo Lin School of Medicine, National University of Singapore, Singapore, Singapore; 3 Ministry of Education and Shanghai Key Laboratory of Children’s Environmental Health, Xinhua Hospital, Shanghai Jiao Tong University School of Medicine, Shanghai, China; 4 School of Public Health, Shanghai Jiao Tong University, Shanghai, China; 5 Department of Biostatistics, Yong Loo Lin School of Medicine, National University of Singapore, Singapore, Singapore; 6 Department of Reproductive Medicine, KK Women’s and Children’s Hospital, Singapore, Singapore; 7 Duke-NUS Medical School, Singapore, Singapore; 8 Department of Obstetrics & Gynaecology and Human Potential Translational Research Programme, Yong Loo Lin School of Medicine, National University of Singapore and National University Health System, Singapore, Singapore; 9 Department of Obstetrics and Gynecology, Lunenfeld-Tanenbaum Research Institute, Mount Sinai Hospital, University of Toronto, Toronto, M5G 1X5 Canada; 10 Department of Paediatrics, KK Women’s and Children’s Hospital, Singapore, Singapore; 11 Lee Kong Chian School of Medicine, Nanyang Technological University, Singapore, Singapore; 12 Department of Maternal Fetal Medicine, KK Women’s and Children’s Hospital, Singapore, Singapore; 13 School of Public Health, Guilin Medical College, Guilin, Guangxi China; 14 Folkhälsan Research Center, Helsinki, Finland; 15 Department of General Practice and Primary Health Care, University of Helsinki, Helsinki, Finland

**Keywords:** Asian, Gestational diabetes mellitus, International Association of Diabetes and Pregnancy Study Groups, World Health Organisation, GUSTO, Shanghai birth cohort

## Abstract

**Background:**

Gestational diabetes mellitus (GDM) has been associated with adverse health outcomes for mothers and offspring. Prevalence of GDM differs by country/region due to ethnicity, lifestyle and diagnostic criteria. We compared GDM rates and risk factors in two Asian cohorts using the 1999 WHO and the International Association of Diabetes and Pregnancy Study Groups (IADPSG) criteria.

**Methods:**

The Shanghai Birth Cohort (SBC) and the Growing Up in Singapore Towards healthy Outcomes (GUSTO) cohort are prospective birth cohorts. Information on sociodemographic characteristics and medical history were collected from interviewer-administered questionnaires. Participants underwent a 2-h 75-g oral glucose tolerance test at 24–28 weeks gestation. Logistic regressions were performed.

**Results:**

Using the 1999 WHO criteria, the prevalence of GDM was higher in GUSTO (20.8%) compared to SBC (16.6%) (*p* = 0.046). Family history of hypertension and alcohol consumption were associated with higher odds of GDM in SBC than in GUSTO cohort while obesity was associated with higher odds of GDM in GUSTO. Using the IADPSG criteria, the prevalence of GDM was 14.3% in SBC versus 12.0% in GUSTO. A history of GDM was associated with higher odds of GDM in GUSTO than in SBC, while being overweight, alcohol consumption and family history of diabetes were associated with higher odds of GDM in SBC.

**Conclusions:**

We observed several differential risk factors of GDM among ethnic Chinese women living in Shanghai and Singapore. These findings might be due to heterogeneity of GDM reflected in diagnostic criteria as well as in unmeasured genetic, lifestyle and environmental factors.

**Supplementary Information:**

The online version contains supplementary material available at 10.1186/s12884-021-04036-5.

## Background

The Developmental Origins of Health and Disease (DoHaD) hypothesis states that exposure to environmental and lifestyle factors during critical window periods in the prenatal, perinatal and early postnatal phases influences the subsequent development of non-communicable diseases in the offspring [1]. Pregnancy is among the most important periods of development, which can be complicated by gestational diabetes mellitus (GDM) characterized by glucose intolerance with the first recognition during pregnancy [2], complicating about 14% of pregnancies globally [3].

GDM has been associated with adverse health outcomes for both mother and child [4]; women with GDM are at increased risk of developing type 2 diabetes mellitus [5], cardiovascular diseases [6] and renal diseases [7] later in life. The hyperglycemic intrauterine environment in GDM has been found to increase the risk of fetal macrosomia and associated fetal complications such as shoulder dystocia, hyperinsulinemia and neonatal morbidities [8]. In addition, babies born to women with GDM have a greater propensity to develop type 2 diabetes mellitus and obesity later in life. These findings highlight the importance of evaluating risk factors and deriving strategies to prevent and treat GDM which may induce epigenetic modifications in utero [9].

Established risk factors for GDM include a previous pregnancy with GDM [10], pre-pregnancy overweight and obesity [11, 12], excessive gestational weight gain [12, 13], advanced maternal age [14], family history of diabetes [14], infant sex, alcohol consumption, family history of hypertension, parity and smoking [15]. Maternal weight gain in early pregnancy that disproportionately consists of increased fat deposition could impact on subsequent maternal insulin resistance [13].

There are global differences in the prevalence of GDM which varies from pooled prevalence of 5.4% to 11.5% in meta-analyses of studies from Europe and Asia, respectively [16, 17] due to differences in factors such as diagnostic criteria, ethnicity, lifestyle, and environmental factors. Given the differences in GDM prevalence between Shanghai and Singapore [18, 19] as well as varying lifestyle and environmental exposures, we sought to compare the rates and risk factors of GDM in two contemporary Asian Chinese cohorts, the Shanghai Birth Cohort (SBC) and the Growing Up in Singapore Towards healthy Outcomes cohort (GUSTO).

## Methods

### Study design and population

#### Shanghai birth cohort

The Shanghai Birth Cohort (SBC) recruited pregnant mothers who sought prenatal care at six obstetric care hospitals in Shanghai, from 2013–2016. Couples who were at least 20 years old, comprised of at least one registered Shanghai resident, intended to obtain prenatal care and deliver at hospitals involved in SBC, lived in Shanghai for at least 2 years and were willing to be involved in the study for at least 2 years were invited to participate [20]. The study protocol was approved by the ethics committee of Shanghai Xinhua Hospital (XHEC-C-2013-001, approved on 7 January 2013) and all participating hospitals. All methods were performed in accordance with the approved guidelines and regulations. All participants gave a written informed consent. In this study, we randomly sampled 1000 out of 3692 participants of Chinese ethnicity who were not receiving chemotherapy or psychotropic drugs from the SBC for comparisons to Chinese participants in the GUSTO cohort so that the selected cohort size is comparable to the GUSTO cohort size.

#### GUSTO cohort

The Growing Up in Singapore Towards healthy Outcomes (GUSTO) cohort study recruited pregnant women attending their first-trimester antenatal dating ultrasound scan clinics at two major public maternity units in Singapore, KK Women’s and Children’s Hospital and National University Hospital from June 2009 to September 2010. Pregnant women aged 18 years and above, from any one of the three major ethnic groups (Chinese, Malay and Indian), who were Singapore citizens or permanent residents and had the intention of delivering in either hospital as well as staying in Singapore for at least the next 5 years, and who had agreed to donate their birth tissues were invited to participate. Women who had type 1 diabetes mellitus, or who were receiving chemotherapy or psychotropic drugs were excluded. Information on sociodemographic characteristics and medical history were collected from interviewer administered questionnaires [21]. The study protocol was approved by the ethics committees of the hospitals involved: SingHealth Centralized Institutional Review Board (2018/2767, approved on 2 March 2019) and the National Healthcare Group Domain Specific Review Board (D/2009/021, approved on 26 February 2009) in Singapore. All methods were performed in accordance with the approved guidelines and regulations. All participants gave written informed consent. In this study, only GUSTO participants of Chinese ethnicity (out of 1247 subjects) were included in the analysis.

#### Subject follow up and assessment of maternal blood glucose concentrations

Participants from SBC were followed up at the recruitment visit (≤17 weeks) and at 24–26 weeks gestation. Questionnaires were administered to collect information on demographics, socio-economic status, lifestyle, obstetric and medical history [20]. Pre-pregnancy weight was self-reported while weight at early pregnancy was measured in the prenatal care clinic. Early pregnancy in SBC was defined as gestational age ≤ 17 weeks so as to include all women who received their first antenatal care in the hospital. Participants underwent a 75-g oral glucose tolerance test (OGTT) at 24–28 weeks’ gestation; fasting (FG), 1-h plasma glucose (1hPG) and 2-h plasma glucose (2hPG) concentrations were obtained using automated biochemical analyzer Hitachi LABOSPECT 008. Information on weight and length of the infant at birth was obtained from hospital medical records.

Participants from GUSTO were followed up at the recruitment visit (< 14 weeks) and at 24–28 weeks of gestation when questionnaires were administered to collect information on demographics, socio-economic status, lifestyle, obstetric and medical history [21]. Pre-pregnancy weight was self-reported while weight at early pregnancy was obtained from case notes. Participants underwent a 75-g OGTT at 24–28 weeks’ gestation; overnight fasting (8–10 h) and 2-h postprandial blood specimens were collected. Colorimetry [Advia 2400 Chemistry system (Siemens Medical Solutions Diagnostics) and Beckman LX20 Pro analyzer (Beckman Coulter)] was used to measure both fasting and 2-h postprandial plasma glucose concentrations. Information on weight and length of the infant at birth obtained from hospital medical records.

Plasma glucose concentrations were used to classify GDM according to the 1999 WHO criteria: ≥7.0 mmol/L for FPG and/or ≥ 7.8 mmol/L for 2hPG in the 2-h 75-g OGTT, and the International Association of Diabetes and Pregnancy Study Groups (IADPSG) criteria: if any one of the plasma glucose values was at or above the following thresholds: 5.1 mmol/L for FPG, 10.0 mmol/L 1hPG and 8.5 mmol/L for 2hPG. Pre-pregnancy body mass index (BMI; kg/m^2^) was calculated as pre-pregnancy weight (kg) divided by height^2^ (m^2^) and categorized as underweight (< 18.5 kg/m^2^), normal weight (18.5–22.9 kg/m^2^), overweight (23.0–27.4 kg/m^2^) and obese (≥ 27.5 kg/m^2^) [22]. Gestational weight gain (GWG) in early pregnancy was defined by weight gain from pre-pregnancy to recruitment visit.

### Statistical analysis

All analyses were performed using SPSS for Windows version 26.0 (SPSS Inc., Chicago, IL, USA) with statistical significance set at 2-sided *p* < 0.05. Descriptive statistics for numerical variables were presented as mean (SD) and n (%) for categorical variables. Differences in numerical variables were assessed using 2 sample *t*-test when normality and homogeneity assumptions were satisfied; otherwise, Mann-Whitney U test was used. Chi-square or Fisher exact test was used for categorical variables. Birthweight percentiles categorization was based on methods described by Mikolajczyk et al. Large and small for gestational age babies were defined >90th and < 10th percentiles, respectively [23]. We standardized GWG in early pregnancy and its velocity (kg/week) into z scores, using BMI category-specific mean and SD values derived from the corresponding study cohort [23]. Predictors of GDM were assessed in logistic regression models for each cohort separately. Interaction effects between predictors were tested in the regression models. We reported odds ratio as prospective data was collected on the prevalence of GDM. The differences across the two cohorts were compared using summarized Z-test. Further analysis was performed in GUSTO cohort by adding citizenship status into the model.

## Results

### Comparison of demographic variables between SBC and GUSTO cohort

**Fig. 1:**
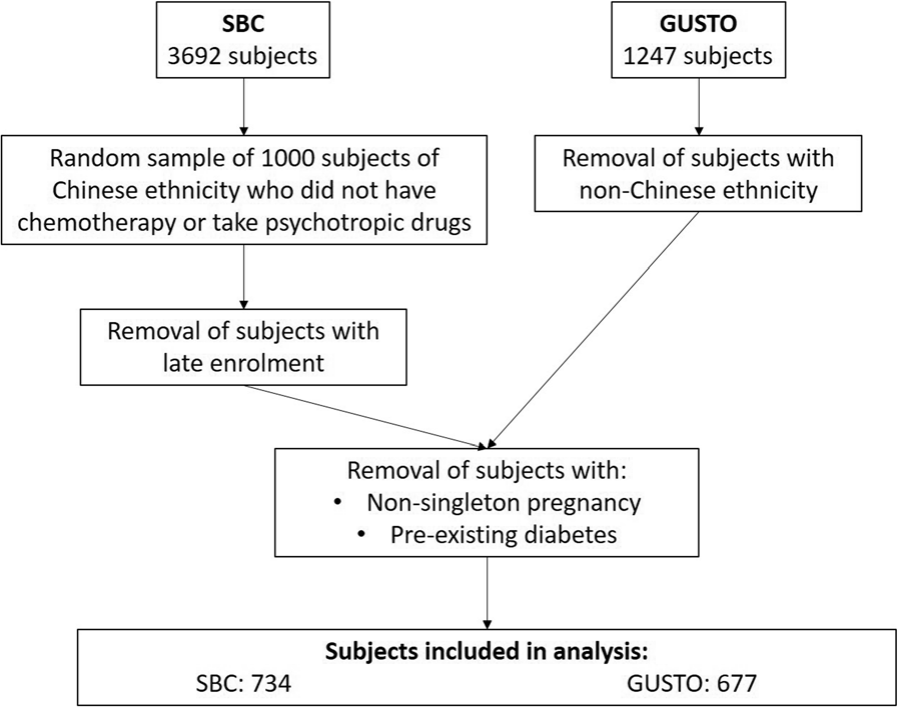
Out of 1000 selected subjects from SBC and 1247 subjects in GUSTO, 734 and 677 respectively were included in the analysis after removal of subjects of non-Chinese ethnicity, with late enrolment, non-singleton pregnancy and pre-existing diabetes

After removal of subjects with late enrolment in the SBC, with non-singleton pregnancy, of non-Chinese ethnicity and with pre-existing diabetes, there were 734 and 677 subjects left in SBC and GUSTO, respectively, in the analysis (Fig. 1). Characteristics of study participants in the two cohorts were presented in Table 1. GUSTO participants had lower gestational age at delivery (38.8 ± 1.4 vs 39.0 ± 1.5 weeks, *p* = 0.002), fasting plasma glucose concentrations (4.3 ± 0.4 vs 4.4 ± 0.4 mmol/L, *p* < 0.001), pre-pregnancy weight (54.5 ± 9.5 vs 56.6 ± 8.7 kg, *p* < 0.001), weight at early pregnancy (56.8 ± 10.3 vs 59.1 ± 9.4 kg, *p* < 0.001), GWG during early pregnancy (1.9 ± 2.4 vs 2.5 ± 3.2 kg, *p* = 0.001), and were shorter (159.1 ± 5.6 vs 162.2 ± 4.6 cm, *p* < 0.001) and older (32.1 ± 4.8 vs 29.8 ± 3.7 years, *p* < 0.001) compared to SBC participants.

**Table 1:** Characteristics of included study participants in SBC and GUSTO cohort

Characteristics	SBC (n = 734)	GUSTO (n = 677)	*P*-value
Maternal age	29.8 ± 3.7	32.1 ± 4.8	< 0.001
Plasma glucose fasting, mmol/L	4.4 ± 0.4	4.3 ± 0.4	< 0.001
Plasma glucose 1 h, mmol/L	7.6 ± 1.6	NA	NA
Plasma glucose 2 h, mmol/L	6.5 ± 1.4	6.6 ± 1.4	0.143
Pre-pregnancy BMI, kg/m^2^	21.5 ± 3.2	21.6 ± 3.4	0.637
BMI at early pregnancy, kg/m^2^	22.5 ± 3.4	22.4 ± 3.7	0.320
Pre-pregnancy weight, kg	56.6 ± 8.7	54.5 ± 9.5	< 0.001
Weight at early pregnancy, kg	59.1 ± 9.4	56.8 ± 10.3	< 0.001
GWG at early pregnancy, kg	2.5 ± 3.2	1.9 ± 2.4	0.001
GWG velocity at early pregnancy, kg/week	0.2 ± 0.2	0.2 ± 0.2	0.911
Height, cm	162.2 ± 4.6	159.1 ± 5.6	< 0.001
Pre-pregnancy BMI (kg/m^2^)			0.369
<18.5(underweight)	109(14.9 %)	82(13.5 %)	
≥ to < 23 (normal)	436(59.4 %)	365(60.2 %)	
≥ to < 27.5 (overweight)	154(21.0 %)	118(19.5 %)	
≥ (obese)	35(4.8 %)	41(6.8 %)	
GWG in early pregnancy (z score)			0.985
<-1	82(11.2 %)	68(11.5 %)	
-1 to 1	552(75.6 %)	446(75.2 %)	
> 1	96(13.2 %)	79(13.3 %)	
Alcohol consumption during pregnancy	2(0.3 %)	21(3.3 %)	< 0.001
Family history of diabetes	62(9.2 %)	165(24.4 %)	< 0.001
Family history of hypertension	225(32.7 %)	279(41.2 %)	0.001
Current or ever smoker	22(3.0 %)	61(9.4 %)	< 0.001
History of GDM in previous pregnancy	3(0.4 %)	22(3.2 %)	< 0.001
Personal history of chronic hypertension	1(0.1 %)	8(1.2 %)	0.017
Parous	84(11.5 %)	316(48.8 %)	< 0.001
Gestational age at delivery, week	39.0 ± 1.5	38.8 ± 1.4	0.002
Male fetus	349(49.7 %)	340(52.6 %)	0.298
Citizenship status	NA		NA
Singapore Citizen born in Singapore		379(56.0 %)	
Converted Citizen or permanent resident		298(44.0 %)	

A higher proportion of GUSTO participants consumed alcohol during pregnancy compared to SBC participants (3.3% versus 0.3%, Table 1), were currently smoking or had ever smoked (9.4% versus 3.0%), had a family history of diabetes (24.4% versus 9.2%), had a family history of hypertension (41.2% versus 32.7%), had a history of GDM in a previous pregnancy (3.2% versus 0.4%) and had a personal history of hypertension (1.2% vs 0.1%) compared to SBC participants. More GUSTO participants were parous compared to SBC participants (48.8% versus 11.5%).

### Comparison of risk factors of GDM between SBC and GUSTO cohort

Using the 1999 WHO criteria, the prevalence of GDM was higher in GUSTO cohort (20.8%) compared to SBC (16.6%) (*p* = 0.046). Using the IADPSG criteria, the prevalence of GDM was 14.3% in SBC (using all three glucose time point measures) versus 12.0% in GUSTO (defined by the fasting and 2 h glucose data only).

### 1999 WHO criteria

Using the 1999 WHO criteria in the SBC, GDM was associated with maternal age (OR 1.1, 95% CI 1.0–1.2, *p* = 0.020) and pre-pregnancy BMI (OR 1.1, 95% CI 1.0–1.2, *p* = 0.028, Table 2). Further analysis with pre-pregnancy BMI categories showed that overweight (OR 2.3, 95% CI 1.3–3.9, *p* = 0.004, Table 3) was associated with GDM. Family history of hypertension and alcohol consumption were associated with higher odds of GDM in SBC than in GUSTO cohort (Tables 2 and 3). The analysis was repeated with the use of GWG velocity z score and similar results were obtained (Supplementary Tables 1 and 2, Additional file 1).

**Table 2:** Associations between risk factors and GDM defined by 1999 WHO criteria in SBC and GUSTO cohort

GDM	Shanghai Birth cohort Unadjusted		GUSTO Birth Cohort Unadjusted		Shanghai Birth cohort Adjusted		GUSTO Birth Cohort Adjusted		P-value*
	OR (95 % CI)	P-value	OR (95 % CI)	P-value	OR (95 % CI)	P- value	OR (95 % CI)	P- value	
Maternal age	1.08(1.03–1.14)	0.003	1.09(1.04–1.14)	< 0.001	1.1(1.0-1.2)	0.020	1.09(1.03–1.14)	0.001	1.000
Pre-pregnancy BMI	1.08(1.03–1.15)	0.005	1.11(1.05–1.17)	< 0.001	1.1(1.0-1.2)	0.028	1.1(1.0-1.2)	0.002	1.000
GWG at early pregnancy (z score)									
<-1	1.0		1.0		1.0		1.0		
-1 to 1	0.96(0.52–1.78)	0.894	1.6(0.8–3.2)	0.208	1.0(0.5–2.2)	0.955	1.7(0.8–3.5)	0.175	0.189
> 1	0.97(0.44–2.13)	0.942	1.8(0.8–4.2)	0.173	0.88(0.34–2.25)	0.786	1.7(0.7-4.0)	0.258	0.211
Alcohol consumption	5.1(0.3–82.0)	0.251	0.67(0.19–2.32)	0.526	8.8(0.5-144.8)	0.128	0.73(0.20–2.64)	0.632	< 0.001
Family history of diabetes	1.6(0.8-3.0)	0.165	1.0(0.6–1.6)	0.981	1.0(0.5–2.1)	0.996	0.86(0.52–1.43)	0.566	0.755
Family history of hypertension	1.6(1.0-2.4)	0.032	0.78(0.53–1.16)	0.223	1.5(0.9–2.5)	0.092	0.67(0.43–1.04)	0.076	0.016
Current or ever smoker	1.1(0.4–3.4)	0.842	0.79(0.39–1.61)	0.515	1.6(0.4-6.0)	0.509	1.1(0.5–2.3)	0.858	0.528
Parous	1.0(0.5–1.8)	1.000	1.2(0.8–1.7)	0.470	0.74(0.34–1.61)	0.443	0.97(0.64–1.49)	0.893	0.610
Male fetus	1.1(0.7–1.7)	0.633	1.1(0.7–1.6)	0.781	1.1(0.7–1.7)	0.801	1.1(0.7–1.6)	0.744	1.000

Adjusted for maternal age, pre-pregnancy BMI, GWG at early pregnancy (z score), alcohol consumption, family history of diabetes, family history of hypertension, smoking status, parity, fetal sex

In the adjusted model, 74.4 % of SBC subjects (546 out of 734) were used, 82.9 % of GUSTO subjects (561 out of 677) were used

*P value for the difference between the two cohorts in the adjusted model

**Table 3:** Effect of GWG among women of different pre-pregnancy BMI and fetal sex on GDM development defined by 1999 WHO criteria

GDM	Shanghai Birth cohort Unadjusted		GUSTO Birth Cohort Unadjusted		Shanghai Birth cohort Adjusted		GUSTO Birth Cohort Adjusted		P-value*
	OR (95 % CI)	P-value	OR (95 % CI)	P-value	OR (95 % CI)	P-value	OR (95 % CI)	P-value	
Maternal age	1.08(1.03–1.14)	0.003	1.09(1.04–1.14)	< 0.001	1.1(1.0-1.2)	0.014	1.08(1.03–1.13)	0.003	1.000
GWG at early pregnancy (z score)	0.86(0.70–1.05)	0.133	1.2(1.0-1.5)	0.085	0.69(0.44–1.08)	0.106	1.2(0.8–1.7)	0.444	0.088
Pre-pregnancy BMI									
< 18.5	0.72(0.37–1.39)	0.324	0.93(0.49–1.80)	0.838	0.85(0.38–1.90)	0.687	1.0(0.5–2.1)	0.911	0.785
≥ 18.5 to < 23	1.0		1.0		1.0		1.0		
≥ 23 to < 27.5	1.8(1.2–2.9)	0.009	1.9(1.1-3.0)	0.014	2.3(1.3–3.9)	0.004	1.7(1.0-2.9)	0.047	0.124
≥ 27.5	2.0(0.9–4.5)	0.088	3.7(1.8–7.3)	< 0.001	1.9(0.7–5.5)	0.239	3.7(1.8–7.8)	< 0.001	0.005
Alcohol consumption	5.1(0.3–82.0)	0.251	0.67(0.19–2.32)	0.526	8.7(0.5-144.1)	0.130	0.75(0.21–2.69)	0.656	< 0.001
Family history of diabetes	1.6(0.8-3.0)	0.165	1.0(0.6–1.6)	0.981	0.99(0.48–2.04)	0.967	0.84(0.50–1.41)	0.512	0.717
Family history of hypertension	1.6(1.0-2.4)	0.032	0.78(0.53–1.16)	0.223	1.5(0.9–2.5)	0.100	0.69(0.43–1.08)	0.103	0.021
Current or ever smoker	1.1(0.4–3.4)	0.842	0.79(0.39–1.61)	0.515	1.8(0.4-7.0)	0.421	1.1(0.5–2.3)	0.880	0.398
Parous	1.0(0.5–1.8)	1.000	1.2(0.8–1.7)	0.470	0.68(0.31–1.50)	0.338	1.0(0.7–1.6)	0.880	0.481
Male fetus	1.1(0.7–1.7)	0.633	1.1(0.7–1.6)	0.781	1.1(0.7–1.7)	0.783	1.0(0.7–1.6)	0.868	0.747

Adjusted for maternal age, GWG at early pregnancy, pre-pregnancy BMI group, alcohol consumption, family history of diabetes, family history of hypertension, smoking status, parity, fetal sex

In the adjusted model, 74.4 % of SBC subjects (546 out of 734) were used, 82.9 % of GUSTO subjects (561 out of 677) were used

*P value for the difference between the two cohorts in the adjusted model

Interaction between GWG and pre pregnancy BMI group and fetal sex were included in the model but not significant

Overall interaction for GWG and pre pregnancy BMI group: p = 0.737 for SBC, p = 0.088 for GUSTO

Interaction for GWG and fetal sex: p = 0.254 for SBC, p = 0.674 for GUSTO

In the GUSTO cohort, GDM was associated with maternal age (OR 1.09, 95% CI 1.03–1.14, *p* = 0.001) and pre-pregnancy BMI (OR 1.1, 95% CI 1.0–1.2, *p* = 0.002, Table 2). Further analysis with pre-pregnancy BMI categories showed that overweight (OR 1.7, 95% CI 1.0–2.9, *p* = 0.047) and obesity (OR 3.7, 95% CI 1.8–7.8, *p* < 0.001, Table 3) were associated with GDM. Obesity was associated with higher odds of GDM in GUSTO than in SBC (Table 3). The analysis was repeated using GWG velocity z score and similar results were obtained (Supplementary Tables 1 and 2, Additional file 1). GWG velocity in early pregnancy was associated with higher odds of development of GDM in GUSTO compared to SBC (*p* = 0.022, Supplementary Table 1, Additional file 1).

Further analysis was performed in GUSTO cohort by adding citizenship status in the model. Using the 1999 WHO criteria, maternal age remained significantly associated with GDM (OR 1.1, 95% CI 1.0–1.2, *p* = 0.003, Supplementary Table 3, Additional file 1). Analysis with pre-pregnancy BMI categories also showed that maternal age remained significantly associated with GDM (OR 1.1, 95% CI 1.0–1.2, p = 0.003) and obesity (OR 12.2, 95% CI 1.6–93.4, *p* = 0.016, Supplementary Table 4, Additional file 1) was associated with GDM. There were no significant interactions between citizenship status and all risk factors (Supplementary Tables 3 and 4, Additional file 1). The analysis was repeated using GWG velocity z score and similar results were obtained (Supplementary Tables 5 and 6, Additional file 1).

### IADPSG criteria

Using the IADPSG criteria in the SBC, GDM was associated with maternal age (OR 1.1, 95% CI 1.0–1.2, *p* = 0.046) and pre-pregnancy BMI (OR 1.14, 95% CI 1.05–1.23, *p* = 0.001, Table 4). Further analysis with pre-pregnancy BMI categories showed that overweight (OR 2.5, 95% CI 1.4–4.4, *p* = 0.002) and obesity (OR 3.6, 95% CI 1.3–9.6, *p* = 0.011, Table 5) were associated with GDM. Being overweight, having family history of diabetes and alcohol consumption were associated with higher odds of GDM in the SBC than in the GUSTO cohort (Tables 4 and 5). The analysis was repeated using GWG velocity z score and similar results were obtained (Supplementary Tables 7 and 8, Additional file 1).

**Table 4:** Associations between risk factors and GDM defined by IADPSG criteria in SBC and GUSTO cohort

GDM	Shanghai Birth cohort Unadjusted		GUSTO Birth Cohort Unadjusted		Shanghai Birth cohort Adjusted		GUSTO Birth Cohort Adjusted		P-value*
	OR (95 % CI)	P-value	OR (95 % CI)	P-value	OR (95 % CI)	P- value	OR (95 % CI)	P- value	
Maternal age	1.1(1.0-1.2)	0.002	1.08(1.03–1.14)	0.004	1.1(1.0-1.2)	0.046	1.06(1.00-1.13)	0.064	1.000
Pre-pregnancy BMI	1.14(1.07–1.21)	< 0.001	1.13(1.06–1.21)	< 0.001	1.14(1.05–1.23)	0.001	1.1(1.0-1.2)	0.006	1.000
GWG at early pregnancy (z score)									
<-1	1.0		1.0		1.0		1.0		
-1 to 1	1.0(0.5-2.0)	0.998	1.2(0.5–2.8)	0.662	1.4(0.6–3.5)	0.403	1.2(0.5–2.8)	0.727	0.750
> 1	1.6(0.7–3.6)	0.260	1.4(0.5–3.8)	0.529	2.1(0.8–5.6)	0.159	1.3(0.5–3.8)	0.616	0.265
Alcohol consumption	5.8(0.4–93.9)	0.214	0.85(0.19–3.72)	0.824	12.0(0.7–198.0)	0.083	0.99(0.22–4.55)	0.988	< 0.001
Family history of diabetes	2.3(1.3–4.3)	0.008	1.3(0.7–2.2)	0.388	1.8(0.9–3.6)	0.089	0.67(0.35–1.30)	0.240	0.020
Family history of hypertension	1.5(1.0-2.3)	0.077	1.1(0.7–1.8)	0.649	1.4(0.8–2.3)	0.262	0.88(0.51–1.54)	0.661	0.182
Current or ever smoker	0.94(0.27–3.25)	0.928	0.70(0.27–1.80)	0.456	1.6(0.4–6.3)	0.507	0.87(0.31–2.42)	0.783	0.405
Parous	1.2(0.7–2.3)	0.519	1.3(0.8–2.2)	0.225	0.75(0.33–1.72)	0.502	0.90(0.52–1.58)	0.717	0.768
History of GDM in previous pregnancy	12.2(1.1-135.3)	0.042	8.3(3.3–20.7)	< 0.001	2.5(0.2–42.7)	0.520	7.7(2.6–22.4)	< 0.001	< 0.001
Male fetus	0.97(0.63–1.49)	0.873	1.0(0.6–1.6)	0.988	1.0(0.6–1.6)	0.862	0.83(0.49–1.41)	0.496	0.644

Adjusted for maternal age, pre-pregnancy BMI, GWG at early pregnancy (z score), alcohol consumption, family history of diabetes, family history of hypertension, smoking status, parity, history of GDM in previous pregnancy, fetal sex

In the adjusted model, 74.4 % of SBC subjects (546 out of 734) were used, and 82.9 % of GUSTO subjects (561 out of 677) were used

*P value for the difference between the two cohorts in the adjusted model

**Table 5:** Effect of GWG among women of different pre-pregnancy BMI and fetal sex on GDM development defined by IADPSG criteria

GDM	Shanghai Birth cohort Unadjusted		GUSTO Birth Cohort Unadjusted		Shanghai Birth cohort Adjusted		GUSTO Birth Cohort Adjusted		P-value*
	OR (95 % CI)	P-value	OR (95 % CI)	P-value	OR (95 % CI)	P-value	OR (95 % CI)	P-value	
Maternal age	1.1(1.0-1.2)	0.002	1.08(1.03–1.14)	0.004	1.1(1.0-1.2)	0.030	1.06(0.99–1.12)	0.099	1.000
GWG at early pregnancy (z score)	1.0(0.8–1.3)	0.690	1.1(0.9–1.5)	0.277	1.1(0.7–1.7)	0.722	1.1(0.7–1.8)	0.611	1.000
Pre-pregnancy BMI									
< 18.5	0.82(0.40–1.67)	0.579	0.58(0.22–1.53)	0.274	1.0(0.4–2.3)	0.972	0.69(0.25–1.89)	0.471	0.650
≥ 18.5 to < 23	1.0		1.0		1.0		1.0		
≥ 23 to < 27.5	2.5(1.5-4.0)	< 0.001	1.5(0.8–2.8)	0.190	2.5(1.4–4.4)	0.002	1.3(0.7–2.6)	0.415	0.007
≥ 27.5	3.7(1.7-8.0)	0.001	4.3(2.0-9.1)	< 0.001	3.6(1.3–9.6)	0.011	3.8(1.7–8.8)	0.001	0.762
Alcohol consumption	5.8(0.4–93.9)	0.214	0.85(0.19–3.72)	0.824	11.8(0.7-196.4)	0.086	1.1(0.2–4.9)	0.939	< 0.001
Family history of diabetes	2.3(1.3–4.3)	0.008	1.3(0.7–2.2)	0.388	1.8(0.9–3.6)	0.081	0.67(0.34–1.32)	0.247	0.022
Family history of hypertension	1.5(1.0-2.3)	0.077	1.1(0.7–1.8)	0.649	1.4(0.8–2.3)	0.250	0.91(0.52–1.60)	0.746	0.213
Current or ever smoker	0.94(0.27–3.25)	0.928	0.70(0.27–1.80)	0.456	1.9(0.5–7.9)	0.373	0.87(0.31–2.44)	0.793	0.241
Parous	1.2(0.7–2.3)	0.519	1.3(0.8–2.2)	0.225	0.68(0.29–1.58)	0.365	0.93(0.53–1.65)	0.810	0.631
History of GDM in previous pregnancy	12.2(1.1-135.3)	0.042	8.3(3.3–20.7)	< 0.001	2.4(0.1–44.6)	0.552	8.3(2.8–24.4)	< 0.001	< 0.001
Male fetus	0.97(0.63–1.49)	0.873	1.0(0.6–1.6)	0.988	0.94(0.56–1.58)	0.822	0.82(0.48–1.42)	0.480	0.754

Adjusted for maternal age, GWG at early pregnancy, pre-pregnancy BMI group, alcohol consumption, family history of diabetes, family history of hypertension, smoking status, parity, history of GDM in previous pregnancy, fetal sex

In the adjusted model, 74.4 % of SBC subjects (546 out of 734) were used while 82.9 % of GUSTO subjects (561 out of 677) were used

*P value refers to the difference between cohorts in the adjusted model

Interaction between GWG and pre pregnancy BMI group and fetal sex was included in the model but not significant

Overall interaction for GWG and pre pregnancy BMI group: p = 0.379 for SBC, p = 0.729 for GUSTO

Interaction for GWG and fetal sex: p = 0.798 for SBC, p = 0.582 for GUSTO

**Supplementary Information**

Additional file 1. Supplementary tables

In the GUSTO cohort, GDM was associated with pre-pregnancy BMI (OR 1.1, 95% CI 1.0–1.2, *p* = 0.006) and history of GDM (OR 7.7, 95% CI 2.6–22.4, *p* < 0.001, Table 4). Further analysis with pre-pregnancy BMI categories showed that obesity (OR 3.8, 95% CI 1.7–8.8, p = 0.001, Table 5) was associated with GDM. The analysis was repeated using GWG velocity z score and similar results were obtained (Supplementary Tables 7 and 8, Additional file 1). A history of GDM was significantly associated with higher odds of GDM in the GUSTO cohort than in the SBC (Tables 4 and 5 and Supplementary Tables 7 and 8, Additional file 1).

There were no significant interactions between GWG or weight gain velocity and pre-pregnancy BMI or fetal sex in relation to the odds of GDM in SBC and GUSTO cohort (Tables 3 and 5 and Supplementary Tables 2 and 8, Additional file 1, all *p* > 0.05).

Further analysis was performed in GUSTO cohort by adding citizenship status in the model. Using the IADPSG criteria, history of GDM in previous pregnancy remained significantly associated with GDM (OR 26.4, 95% CI 2.6–269.8, p = 0.006, Supplementary Table 9, Additional file 1). Similarly, analysis using pre-pregnancy BMI categories also showed that history of GDM in previous pregnancy remained significantly associated with GDM (OR 37.1, 95% CI 3.3–416.5, *p* = 0.003). Obesity (OR 11.6, 95% CI 1.3–102.4, *p* = 0.027) was also associated with GDM (Supplementary Table 10, Additional file 1). There were no significant interactions between citizenship status and all risk factors (Supplementary Tables 9 and 10, Additional file 1). The analysis was repeated using GWG velocity z -score and similar results were obtained (Supplementary Tables 11 and 12, Additional file 1).

### Preterm birth and birthweight for gestational age

Using the 1999 WHO criteria, preterm birth rates were non-significantly higher among women with GDM (8.3%) vs. without (4.4%) in the SBC (*p* = 0.077, Supplementary Table 13, Additional file 1), but were significantly higher (12.1% vs 6.1%) in the GUSTO cohort (*p* = 0.018). There were no significant differences in birthweights nor the rates of small or large for gestational age births in GDM and non-GDM groups in both cohorts (Supplementary Table 13, Additional file 1).

Using the IADPSG criteria, there were no significant differences in the rates of preterm birth, large or small for gestational age births in GDM and non-GDM groups in both cohorts (Supplementary Table 14, Additional file 1).

## Discussion

In this study, we compared risk factors of GDM in two Chinese cohorts in Shanghai and Singapore. To the best of our knowledge, this is the first study comparing risk factors of GDM in ethnic Chinese living in different geographical locations. The study findings will primarily be discussed in relation to IADPSG criteria.

We found maternal age and pre-pregnancy BMI as common associated factors of GDM diagnosed by WHO 1999 and IADPSG criteria in both cohorts. Family history of diabetes and being overweight were associated with higher odds of GDM diagnosed by IADPSG criteria in SBC than in GUSTO.

Maternal age is a well-documented risk factor of GDM. In a meta-analysis involving 24 studies and over 120 million participants, GDM risk was demonstrated to increase linearly with age [24]. The relative risk of GDM in an Asian population was estimated to increase by 13% for every year increase in maternal age over 18 years. Another study of pregnant women from 18 cities in China also found advanced maternal age to be a strong predictor of GDM risk [25]. This could be attributed to an increase in dysfunctional preadipocytes and ectopic fat redistribution with aging which may cause lipotoxicity with the release of proinflammatory cytokines and chemokines disrupting insulin signaling [26, 27].

As expected, higher pre-pregnancy BMI was associated with increased odds of GDM in both cohorts. A meta-analysis of 70 studies involving over 600,000 women showed that each 1 kg/m^2^ increase in BMI is linked to a 0.92% increase in GDM prevalence [28]. Similar to aging, obesity is associated with insulin resistance, ectopic fat accumulation and chronic inflammation as adipocytes release proinflammatory cytokines and chemokines [29]. Further analysis by classifying pre-pregnancy BMI into categories defined for Asian metabolic risk showed that BMI  ≥27.5 kg/m^2^ (obese) was associated with increased odds of GDM [30]. Studies have shown that GDM risk increases linearly with pre-pregnancy BMI [11, 28]. A study of Arab women found a significant association between obesity (prepregnancy BMI ≥30 kg/m^2^) and GDM [31]. Tsiotra et al. reported that obese women had higher levels of circulating adipokines such as chemerin and leptin that may be responsible for inflammation and insulin resistance [32].

Family history of diabetes was associated with significantly higher odds of GDM in the SBC. A meta-analysis involving 84 studies in Asia also found a significant positive association between family history of diabetes and GDM [17]. This may be explained by genetic predisposition as pancreatic islet β-cell function and/or abnormalities can be inherited through single nucleotide polymorphisms (SNPs) in genes linked to insulin signaling or secretion [33]. Although the prevalence of diabetes in family members was lower in SBC than GUSTO, the higher odds of association with GDM in the SBC may be due to several reasons; genetic differences such as the rs10229583 gene locus polymorphism near PAX4 gene that is responsible for the development of pancreatic β cells was implicated in earlier age of diagnosis of type 2 diabetes mellitus in Chinese population in Shanghai and Hong Kong but the effect was less prominent in Singapore Chinese [34]. The risk allele frequency of most SNPs responsible for type 2 diabetes mellitus was also lower in East Asians as compared to South Asians, suggesting ethnic differences even within the Asian population [35].

We did not observe any significant association between GWG in early pregnancy and risk of GDM in the two cohorts. This is supported by Ruhstaller et al. who also reported no association between GDM and weight gain during the first 20 weeks of pregnancy with adjustment for pre-pregnancy BMI [36]. Interestingly, GWG velocity in early pregnancy was associated with significantly higher odds of GDM in GUSTO cohort than in SBC using the 1999 WHO criteria. A possible reason could be due to modulation of GDM risk by genetic variants in fat mass and obesity associated gene such rs1121980 that has been reported to lower the risk of GDM in Chinese in China while not affecting the risk of type 2 diabetes in Singaporean Chinese [37, 38]. Hence differences in genetic make-up may have modulated the impact of GWG on GDM risk in SBC and GUSTO.

Epigenetic changes induced by lifestyle may also explain the difference in risk between the two cohorts. Poorer sleep quality alters DNA methylation in adipose tissues, resulting in higher fat accumulation [39] and risk of GDM [40]. Studies utilizing the Pittsburgh Sleep Quality Index showed that Singaporeans had worse sleep quality with mean score of 5.08 to 5.51 [41] as compared to Shanghai residents with a mean score of 3.69 [42]. A low consumption of folate, a methyl donor, is also linked to obesity and insulin resistance [43]. Chinese women from Singapore had lower plasma folate concentration than those from Shanghai [44, 45].

We observed that being overweight was also associated with higher odds of GDM in SBC as compared to GUSTO. This may be due to lifestyle differences. For example, participants from China and Singapore have different dietary patterns which may have affected risk of developing GDM. A study from China reported that the dietary patterns of pregnant women can be classified into Western (fried/baked food, white meat and dairy), traditional (fine grain, red meat, light-coloured vegetables and tubers), mixed (red meat, shrimp or shellfish and edible fungi) and prudent (deep-sea fish and dark-coloured vegetables) where Western and traditional patterns increased the risk of GDM [46]. Conversely, a study from Singapore reported that the dietary patterns of pregnant women can be classified into vegetable-fruit-rice-based, seafood-noodle-based and pasta-cheese-processed-meat where a seafood-noodle-based diet reduced the risk of GDM [47]. In addition, women in China tend to have lower levels of physical activity during pregnancy due to traditional customs where pregnant women are regarded as vulnerable which may have increased the risk of GDM in high-risk women [48].

We also did not observe any differences in odds of GDM between Singaporean citizens and Chinese immigrants from the GUSTO cohort, suggesting that environmental factors play an important role in the development of GDM.

While studies reported higher risks of preterm [49], macrosomia [8] and high birthweight [50] births in GDM, we did not observe these associations in SBC and GUSTO cohorts. This may be explained by effective dietary interventions as women in both cohorts attended prenatal care in high-quality tertiary care centers. Vally et al. showed that GDM mothers with well-controlled diet and glycemia did not have an increased risk of macrosomia [51]. A meta-analysis of studies on Chinese women found that low GI or fiber-enriched diets reduced the risk of preterm births [52].

In this study, we compared 2 different diagnostic criteria of GDM. The IADPSG criteria detects more GDM cases as compared to other criteria [53]. The WHO 1999 criteria might miss GDM cases with elevated fasting glucose concentrations [54].

GDM is known to be a heterogeneous condition and the two different diagnostic criteria probably identify different subtypes of GDM. Supporting evidence on GDM subtypes is provided by the observation of different clinical outcomes associated with elevated fasting and postprandial glucose levels [55]. Women with elevated fasting and normal postprandial values had a higher risk of having large for gestational age babies while women with normal fasting and elevated postprandial values had a higher risk of preterm delivery and gestational hypertension as compared to women without GDM [55].

Strengths of the study include extensive data collection and regular follow up of the subjects in both studies. This is the first study comparing GDM prevalence and risk factors in two cohorts of Chinese women from different countries, namely China and Singapore, using two GDM diagnostic criteria. It is well-known that global differences in GDM prevalence exist due to differences in factors such as diagnostic criteria, ethnicity, lifestyle, and environmental factors. This novel study helps to elucidate if diagnostic criteria or lifestyle differences account for the differences in GDM rates in Chinese people. A limitation of the study is the absence of collection of blood glucose data at the 1 h timepoint in GUSTO which may have contributed to the lower rates of GDM using the IADPSG criteria and misclassification of some women with GDM as non-GDM, hence diluting the observed risks [56]. Nevertheless, we observed similar findings such as pre-pregnancy BMI being associated with increased odds of GDM using both the WHO 1999 and IADPSG criteria. Another limitation is the inability to assess nutritional aspects and environmental factors such as air pollution which may have explained the differences in risk factors and prevalence of GDM in SBC and GUSTO. We also relied on self-reported pre-pregnancy body weight in this study which is subjected to recall bias.

The findings of the study help to provide different clinical recommendations for GDM prevention in Chinese women from China and Singapore. Women who are intending to start a family can be advised to conceive at a younger age and to control their pre-pregnancy weight. Native Chinese women residing in China who have family history of diabetes and/or are overweight can also be identified as a high-risk group and followed more closely throughout their pregnancy for timely interventions against GDM.

## Conclusions

In conclusion, we observed some differential risk factors of GDM among ethnic Chinese women living in Shanghai and Singapore. These findings might be due to heterogeneity of GDM reflected in diagnostic criteria as well as in unmeasured genetic, lifestyle and environmental factors.

## Supplementary Information

**Additional file 1.** Supplementary tables.

## Data Availability

The datasets used and/or analysed during the current study are available from the corresponding author on reasonable request.

## Abbreviations

BMI: Body mass index
DoHaD: Developmental Origins of Health and Disease
FG: Fasting glucose
GDM: Gestational diabetes mellitus
GUSTO: Growing Up in Singapore Towards healthy Outcomes
GWG: Gestational weight gain
IADPSG: International Association of Diabetes and Pregnancy Groups
OGTT: Oral glucose tolerance test
PG: Plasma glucose
SBC: Shanghai Birth Cohort
SNPs: Single nucleotide polymorphisms
