# Supplementary material for: Comparative epidemiology of gestational diabetes in ethnic Chinese from Shanghai birth cohort and growing up in Singapore towards healthy outcomes cohort
Source: BMC Pregnancy Childbirth. 2021 Aug 18;21:566. doi: 10.1186/s12884-021-04036-5 (PMC8375167; doi:10.1186/s12884-021-04036-5)
Supplement: Supplementary file 1 — Additional file 1. Supplementary tables. [file 12884_2021_4036_MOESM1_ESM.docx]

**Comparative epidemiology of gestational diabetes in ethnic Chinese from Shanghai Birth Cohort and Growing Up in Singapore Towards healthy Outcomes cohort**

Evelyn Xiu Ling Loo^1,2^*, Yuqing Zhang^3,4^*, Qai Ven Yap^5^, Guoqi Yu^3^, Shu E Soh^2^, See Ling Loy^1,6,7^, Hui Xing Lau^1^, Shiao Yng Chan^1,8^, Lynette Pei Chi Shek^2^, Zhong-Cheng Luo^3,9^, Fabian Kok Peng Yap^7,10,11^, Kok Hian Tan^12^, Yap Seng Chong^1,8^, Jun Zhang^3,13#^, Johan Gunnar Eriksson^1,8,14,15#^

1. Singapore Institute for Clinical Sciences (SICS), Agency for Science, Technology and Research (A*STAR), Singapore
2. Department of Paediatrics, Yong Loo Lin School of Medicine, National University of Singapore, Singapore
3. Ministry of Education and Shanghai Key Laboratory of Children’s Environmental Health, Xinhua Hospital, Shanghai Jiao Tong University School of Medicine, Shanghai, China
4. School of Public Health, Shanghai Jiao Tong University, Shanghai, China
5. Department of Biostatistics, Yong Loo Lin School of Medicine, National University of Singapore, Singapore
6. Department of Reproductive Medicine, KK Women's and Children's Hospital, Singapore, Singapore
7. Duke-NUS Medical School, Singapore
8. Department of Obstetrics & Gynaecology and Human Potential Translational Research Programme, Yong Loo Lin School of Medicine, National University of Singapore and National University Health System, Singapore
9. Department of Obstetrics and Gynecology, Lunenfeld-Tanenbaum Research Institute, Mount Sinai Hospital, University of Toronto, Toronto, Canada, M5G 1X5
10. Department of Paediatrics, KK Women's and Children's Hospital
11. Lee Kong Chian School of Medicine, Nanyang Technological University, Singapore
12. Department of Maternal Fetal Medicine, KK Women’s and Children’s Hospital (KKWCH), Singapore
13. School of Public Health, Guilin Medical College, Guangxi, China
14. Folkhälsan Research Center, Helsinki, Finland
15. Department of General Practice and Primary Health Care, University of Helsinki, Finland

*joint first authors

#joint senior authors

Word count of main text: 3986

**Correspondence: Evelyn Loo, Singapore Institute for Clinical Sciences, Brenner Centre for Molecular Medicine, 30 Medical Drive, Singapore 117609. Email address:** [**evelyn_loo@sics.a-star.edu.sg**](mailto:evelyn_loo@sics.a-star.edu.sg)

Supplementary Table 1. Associations between risk factors and GDM defined by 1999 WHO criteria (using GWG velocity z score).

| GDM | Shanghai Birth cohort  Unadjusted | | GUSTO Birth Cohort  Unadjusted | | Shanghai Birth cohort  Adjusted | | GUSTO Birth Cohort  Adjusted | | P-value* |
| --- | --- | --- | --- | --- | --- | --- | --- | --- | --- |
|  | OR (95% CI) | P-value | OR (95% CI) | P-value | OR (95% CI) | P- value | OR (95% CI) | P- value |  |
| Maternal age | 1.08(1.03-1.14) | 0.003 | 1.09(1.04-1.14) | <0.001 | 1.1(1.0-1.2) | 0.014 | 1.08(1.03-1.14) | 0.001 | 1.000 |
| Pre-pregnancy BMI | 1.08(1.03-1.15) | 0.005 | 1.11(1.05-1.17) | <0.001 | 1.1(1.0-1.2) | 0.030 | 1.1(1.0-1.2) | 0.002 | 1.000 |
| GWG velocity in early pregnancy (z score) | 0.84(0.68-1.03) | 0.092 | 1.2(1.0-1.5) | 0.042 | 0.83(0.65-1.06) | 0.140 | 1.2(1.0-1.5) | 0.109 | 0.022 |
| Alcohol consumption | 5.1(0.3-82.0) | 0.251 | 0.67(0.19-2.32) | 0.526 | 8.6(0.5-141.9) | 0.131 | 0.75(0.21-2.70) | 0.654 | <0.001 |
| Family history of diabetes | 1.6(0.8-3.0) | 0.165 | 1.0(0.6-1.6) | 0.981 | 0.99(0.48-2.05) | 0.972 | 0.87(0.53-1.45) | 0.602 | 0.790 |
| Family history of hypertension | 1.6(1.0-2.4) | 0.032 | 0.78(0.53-1.16) | 0.223 | 1.6(0.9-2.6) | 0.081 | 0.68(0.44-1.07) | 0.096 | 0.009 |
| Current or ever smoker | 1.1(0.4-3.4) | 0.842 | 0.79(0.39-1.61) | 0.515 | 1.7(0.4-6.5) | 0.435 | 1.0(0.5-2.3) | 0.917 | 0.388 |
| Parous | 1.0(0.5-1.8) | 1.000 | 1.2(0.8-1.7) | 0.470 | 0.73(0.33-1.59) | 0.426 | 0.99(0.65-1.52) | 0.972 | 0.568 |
| Male fetus | 1.1(0.7-1.7) | 0.633 | 1.1(0.7-1.6) | 0.781 | 1.1(0.7-1.7) | 0.824 | 1.1(0.7-1.6) | 0.747 | 1.000 |

Adjusted for maternal age, pre-pregnancy BMI, GWG velocity in early pregnancy (z score), alcohol consumption, family history of diabetes, family history of hypertension, smoking status, parity, fetal sex.

In the adjusted model, 74.4% of SBC subjects (546 out of 734) were used, and 82.9% of GUSTO subjects (561 out of 677) were used.

*P value for the difference between the two cohorts in the adjusted model.

Supplementary Table 2. Effect of GWG velocity among women of different pre-pregnancy BMI and fetal sex on GDM development defined by 1999 WHO criteria.

| GDM | Shanghai Birth cohort  Unadjusted | | GUSTO Birth Cohort  Unadjusted | | Shanghai Birth cohort  Adjusted | | GUSTO Birth Cohort  Adjusted | | P-value* |
| --- | --- | --- | --- | --- | --- | --- | --- | --- | --- |
|  | OR (95% CI) | P-value | OR (95% CI) | P-value | OR (95% CI) | P-value | OR (95% CI) | P-value |  |
| Maternal age | 1.08(1.03-1.14) | 0.003 | 1.09(1.04-1.14) | <0.001 | 1.1(1.0-1.2) | 0.013 | 1.08(1.03-1.14) | 0.002 | 1.000 |
| GWG velocity in early pregnancy (z score) | 0.84(0.68-1.03) | 0.092 | 1.2(1.0-1.5) | 0.042 | 0.65(0.41-1.05) | 0.079 | 0.67(0.33-1.37) | 0.276 | 0.963 |
| Pre-pregnancy BMI |  |  |  |  |  |  |  |  |  |
| <18.5 | 0.72(0.37-1.39) | 0.324 | 0.93(0.49-1.80) | 0.838 | 0.86(0.38-1.92) | 0.707 | 1.1(0.5-2.2) | 0.832 | 0.668 |
| ≥18.5 to <23 | 1.0 |  | 1.0 |  | 1.0 |  | 1.0 |  |  |
| ≥23 to <27.5 | 1.8(1.2-2.9) | 0.009 | 1.9(1.1-3.0) | 0.014 | 2.3(1.3-3.9) | 0.003 | 1.7(1.0-2.9) | 0.051 | 0.124 |
| ≥27.5 | 2.0(0.9-4.5) | 0.088 | 3.7(1.8-7.3) | <0.001 | 1.9(0.7-5.5) | 0.218 | 3.7(1.8-7.8) | <0.001 | 0.005 |
| Alcohol consumption | 5.1(0.3-82.0) | 0.251 | 0.67(0.19-2.32) | 0.526 | 8.6(0.5-141.6) | 0.134 | 0.77(0.21-2.78) | 0.689 | <0.001 |
| Family history of diabetes | 1.6(0.8-3.0) | 0.165 | 1.0(0.6-1.6) | 0.981 | 0.98(0.47-2.02) | 0.945 | 0.85(0.51-1.42) | 0.527 | 0.775 |
| Family history of hypertension | 1.6(1.0-2.4) | 0.032 | 0.78(0.53-1.16) | 0.223 | 1.5(0.9-2.5) | 0.097 | 0.69(0.44-1.09) | 0.114 | 0.020 |
| Current or ever smoker | 1.1(0.4-3.4) | 0.842 | 0.79(0.39-1.61) | 0.515 | 1.8(0.5-7.1) | 0.405 | 1.0(0.5-2.3) | 0.926 | 0.306 |
| Parous | 1.0(0.5-1.8) | 1.000 | 1.2(0.8-1.7) | 0.470 | 0.68(0.30-1.50) | 0.335 | 1.0(0.7-1.6) | 0.861 | 0.488 |
| Male fetus | 1.1(0.7-1.7) | 0.633 | 1.1(0.7-1.6) | 0.781 | 1.1(0.7-1.8) | 0.778 | 1.0(0.7-1.6) | 0.837 | 0.755 |

Adjusted for maternal age, GWG velocity in early pregnancy, pre-pregnancy BMI group, alcohol consumption, family history of diabetes, family history of hypertension, smoking status, parity, fetal sex.

In the adjusted model, 74.4% of SBC subjects (546 out of 734) were used, 82.9% of GUSTO subjects (561 out of 677) were used.

*P value for the difference between the two cohorts in the adjusted model.

Interaction between GWG velocity and pre pregnancy BMI group and fetal sex were included in the model but not significant.

Overall interaction for GWG velocity and pre pregnancy BMI group: p=0.791 for SBC, p=0.113 for GUSTO

Interaction for GWG velocity and fetal sex: p=0.262 for SBC, p=0.739 for GUSTO

Supplementary Table 3. Effect of all risk factors among women of different citizenship status on GDM development defined by 1999 WHO criteria in GUSTO cohort.

| GDM | Unadjusted |  | Adjusted | |
| --- | --- | --- | --- | --- |
|  | OR (95% CI) | P-value | OR (95% CI) | P- value |
| Maternal age | 1.09(1.04-1.14) | <0.001 | 1.1(1.0 – 1.2) | 0.003 |
| Pre-pregnancy BMI | 1.11(1.05-1.17) | <0.001 | 1.1(1.0 – 1.2) | 0.085 |
| GWG at early pregnancy (z score) |  |  |  |  |
| <-1 | 1.0 |  | 1.0 |  |
| -1 to 1 | 1.6(0.8-3.2) | 0.208 | 1.2(0.5 – 3.0) | 0.651 |
| >1 | 1.8(0.8-4.2) | 0.173 | 1.1(0.4 – 3.4) | 0.844 |
| Alcohol consumption | 0.67(0.19-2.32) | 0.526 | 0.97(0.18 – 5.27) | 0.975 |
| Family history of diabetes | 1.0(0.6-1.6) | 0.981 | 0.85(0.38 – 1.87) | 0.681 |
| Family history of hypertension | 0.78(0.53-1.16) | 0.223 | 0.72(0.37 – 1.42) | 0.346 |
| Current or ever smoker | 0.79(0.39-1.61) | 0.515 | 2.0(0.6 – 6.7) | 0.243 |
| Parous | 1.2(0.8-1.7) | 0.470 | 0.94(0.50 – 1.75) | 0.839 |
| Male fetus | 1.1(0.7-1.6) | 0.781 | 1.2(0.7 – 2.3) | 0.533 |
| Citizenship status | 0.81(0.55 – 1.19) | 0.289 | 2.7(0 – 260.8) | 0.666 |

Adjusted for maternal age, pre-pregnancy BMI, GWG at early pregnancy (z score), alcohol consumption, family history of diabetes, family history of hypertension, smoking status, parity, fetal sex and citizenship status

In the adjusted model, 82.9% of GUSTO subjects (561 out of 677) were used.

Interaction between Citizenship status and all risk factors were included in the model but not significant.

Overall interaction for immigration status and GWG at early pregnancy: p=0.352.

Interaction for citizenship status and maternal age, pre-pregnancy BMI, alcohol consumption, family history of diabetes, family history of hypertension, smoking status, parity and fetal sex: p=0.120, p=0.732, p=0.566, p=0.825, p=0.799, p=0.137, p=0.853 and p=0.619, respectively.

Supplementary Table 4. Effect of weight gain among women of different pre-pregnancy BMI, citizenship status on GDM development defined by 1999 WHO criteria in GUSTO cohort.

| GDM | Unadjusted | | Adjusted | |
| --- | --- | --- | --- | --- |
|  | OR (95% CI) | P-value | OR (95% CI) | P- value |
| Maternal age | 1.09(1.04-1.14) | <0.001 | 1.1(1.0 – 1.2) | 0.003 |
| GWG at early pregnancy (z score) | 1.2(1.0-1.5) | 0.085 | 0.59(0.26 – 1.34) | 0.205 |
| Pre-pregnancy BMI |  |  |  |  |
| <18.5 | 1.0 |  | 1.0 |  |
| ≥18.5 to <23 | 1.1(0.6 – 2.1) | 0.838 | 1.8(0.5 – 5.9) | 0.364 |
| ≥23 to <27.5 | 2.0(1.0 – 4.1) | 0.063 | 1.6(0.4 – 6.6) | 0.495 |
| ≥27.5 | 3.9(1.6 – 9.4) | 0.002 | 12.2(1.6 – 93.4) | 0.016 |
| Alcohol consumption | 0.67(0.19-2.32) | 0.526 | 0.97(0.17 – 5.45) | 0.969 |
| Family history of diabetes | 1.0(0.6-1.6) | 0.981 | 0.65(0.28 – 1.53) | 0.326 |
| Family history of hypertension | 0.78(0.53-1.16) | 0.223 | 0.78(0.39 – 1.54) | 0.472 |
| Current or ever smoker | 0.79(0.39-1.61) | 0.515 | 1.9(0.6 – 6.3) | 0.286 |
| Parous | 1.2(0.8-1.7) | 0.470 | 0.92(0.48 – 1.75) | 0.797 |
| Male fetus | 1.1(0.7-1.6) | 0.781 | 1.2(0.6 – 2.4) | 0.518 |
| Citizenship status | 0.81(0.55 – 1.19) | 0.289 | 27.5(0.8 – 1006.1) | 0.071 |

Adjusted for maternal age, GWG at early pregnancy, pre-pregnancy BMI group, alcohol consumption, family history of diabetes, family history of hypertension, smoking status, parity, fetal sex and citizenship status.

In the adjusted model, 82.9% of GUSTO subjects (561 out of 677) were used.

Interaction between GWG and pre pregnancy BMI group and fetal sex, interaction between citizenship status and all risk factors were included in the model but not significant.

Overall interaction for GWG and pre pregnancy BMI group: p=0.115.

Interaction for GWG and fetal sex: p=0.804.

Overall interaction for citizenship status and pre pregnancy BMI group: p=0.116.

Interaction for Singapore citizenship status and maternal age, GWG, alcohol consumption, family history of diabetes, family history of hypertension, smoking status, parity, fetal sex: p=0.128, p=0.899, p=0.559, p=0.680, p=0.743, p=0.189, p=0.666 and p=0.578, respectively.

Supplementary Table 5. Effect of all risk factors (using GWG velocity z score) among women of different citizenship status on GDM development defined by 1999 WHO criteria in GUSTO cohort.

| GDM | Unadjusted |  | Adjusted | |
| --- | --- | --- | --- | --- |
|  | OR (95% CI) | P-value | OR (95% CI) | P- value |
| Maternal age | 1.09(1.04-1.14) | <0.001 | 1.1(1.0 – 1.2) | 0.004 |
| Pre-pregnancy BMI | 1.11(1.05-1.17) | <0.001 | 1.1(1.0 – 1.2) | 0.073 |
| GWG velocity in early pregnancy (z score) | 1.2(1.0-1.5) | 0.042 | 1.2(0.9 – 1.5) | 0.319 |
| Alcohol consumption | 0.67(0.19-2.32) | 0.526 | 1.0(0.2 – 5.5) | 0.987 |
| Family history of diabetes | 1.0(0.6-1.6) | 0.981 | 0.84(0.38 – 1.85) | 0.659 |
| Family history of hypertension | 0.78(0.53-1.16) | 0.223 | 0.75(0.38 – 1.47) | 0.397 |
| Current or ever smoker | 0.79(0.39-1.61) | 0.515 | 2.0(0.6 – 6.7) | 0.243 |
| Parous | 1.2(0.8-1.7) | 0.470 | 0.94(0.50 – 1.77) | 0.856 |
| Male fetus | 1.1(0.7-1.6) | 0.781 | 1.2(0.6 – 2.2) | 0.603 |
| Citizenship status | 0.81(0.55 – 1.19) | 0.289 | 9.9(0.1 – 658.4) | 0.284 |

Adjusted for maternal age, pre-pregnancy BMI, GWG velocity in early pregnancy (z score), alcohol consumption, family history of diabetes, family history of hypertension, smoking status, parity, fetal sex and citizenship status

In the adjusted model, 82.9% of GUSTO subjects (561 out of 677) were used.

Interaction between citizenship status and all risk factors were included in the model but not significant.

Interaction for citizenship status and maternal age, pre-pregnancy BMI, GWG velocity, alcohol consumption, family history of diabetes, family history of hypertension, smoking status, parity and fetal sex: p=0.150, p=0.917, p=0.751, p=0.597, p=0.953, p=0.787, p=0.140, p=0.788 and p=0.688, respectively.

Supplementary Table 6. Effect of GWG velocity among women of different pre-pregnancy BMI and citizenship status on GDM development defined by 1999 WHO criteria in GUSTO cohort.

| GDM | Unadjusted | | Adjusted | |
| --- | --- | --- | --- | --- |
|  | OR (95% CI) | P-value | OR (95% CI) | P- value |
| Maternal age | 1.09(1.04-1.14) | <0.001 | 1.1(1.0 – 1.2) | 0.003 |
| GWG velocity in early pregnancy (z score) | 1.2(1.0-1.5) | 0.085 | 0.71(0.32 – 1.55) | 0.387 |
| Pre-pregnancy BMI |  |  |  |  |
| <18.5 | 1.0 |  | 1.0 |  |
| ≥18.5 to <23 | 1.1(0.6 – 2.1) | 0.838 | 1.7(0.5 – 5.4) | 0.389 |
| ≥23 to <27.5 | 2.0(1.0 – 4.1) | 0.063 | 1.6(0.4 – 6.2) | 0.512 |
| ≥27.5 | 3.9(1.6 – 9.4) | 0.002 | 11.5(1.6 – 84.3) | 0.017 |
| Alcohol consumption | 0.67(0.19-2.32) | 0.526 | 0.98(0.17 – 5.54) | 0.982 |
| Family history of diabetes | 1.0(0.6-1.6) | 0.981 | 0.67(0.29 – 1.57) | 0.355 |
| Family history of hypertension | 0.78(0.53-1.16) | 0.223 | 0.79(0.40 – 1.57) | 0.500 |
| Current or ever smoker | 0.79(0.39-1.61) | 0.515 | 1.9(0.6 – 6.3) | 0.296 |
| Parous | 1.2(0.8-1.7) | 0.470 | 0.94(0.49 – 1.79) | 0.845 |
| Male fetus | 1.1(0.7-1.6) | 0.781 | 1.2(0.6 – 2.4) | 0.511 |
| Citizenship Status | 0.81(0.55 – 1.19) | 0.289 | 25.2(0.7 – 908.6) | 0.077 |

Adjusted for maternal age, GWG velocity in early pregnancy, pre-pregnancy BMI group, alcohol consumption, family history of diabetes, family history of hypertension, smoking status, parity, fetal sex and citizenship status.

In the adjusted model, 82.9% of GUSTO subjects (561 out of 677) were used.

Interaction between GWG velocity and pre pregnancy BMI group and fetal sex, interaction between citizenship status and all risk factors were included in the model but not significant.

Overall interaction for GWG velocity and pre pregnancy BMI group: p=0.162.

Interaction for GWG velocity and fetal sex: p=0.938.

Overall interaction for citizenship status and pre pregnancy BMI group: p=0.124.

Interaction for citizenship status and maternal age, GWG velocity, alcohol consumption, family history of diabetes, family history of hypertension, smoking status, parity, fetal sex: p=0.140, p=0.917, p=0.579, p=0.712, p=0.724, p=0.180, p=0.704 and p=0.591, respectively.

Supplementary Table 7. Associations between risk factors and GDM defined by IADPSG criteria (using GWG velocity z score).

| GDM | Shanghai Birth cohort  Unadjusted | | GUSTO Birth Cohort  Unadjusted | | Shanghai Birth cohort  Adjusted | | GUSTO Birth Cohort  Adjusted | | P-value* |
| --- | --- | --- | --- | --- | --- | --- | --- | --- | --- |
|  | OR (95% CI) | P-value | OR (95% CI) | P-value | OR (95% CI) | P- value | OR (95% CI) | P- value |  |
| Maternal age | 1.1(1.0-1.2) | 0.002 | 1.08(1.03-1.14) | 0.004 | 1.1(1.0-1.2) | 0.042 | 1.06(1.00-1.13) | 0.070 | 1.000 |
| Pre-pregnancy BMI | 1.14(1.07-1.21) | <0.001 | 1.13(1.06-1.21) | <0.001 | 1.1(1.1-1.2) | 0.001 | 1.1(1.0-1.2) | 0.005 | 1.000 |
| GWG velocity in early pregnancy (z score) | 1.1(0.9-1.3) | 0.476 | 1.2(0.9-1.5) | 0.225 | 1.1(0.9-1.4) | 0.289 | 1.2(0.9-1.5) | 0.300 | 0.562 |
| Alcohol consumption | 5.8(0.4-93.9) | 0.214 | 0.85(0.19-3.72) | 0.824 | 12.3(0.7-203.4) | 0.079 | 1.0(0.2-4.6) | 0.991 | <0.001 |
| Family history of diabetes | 2.3(1.3-4.3) | 0.008 | 1.3(0.7-2.2) | 0.388 | 1.8(0.9-3.5) | 0.090 | 0.67(0.35-1.31) | 0.242 | 0.019 |
| Family history of hypertension | 1.5(1.0-2.3) | 0.077 | 1.1(0.7-1.8) | 0.649 | 1.4(0.8-2.3) | 0.261 | 0.90(0.51-1.58) | 0.713 | 0.205 |
| Current or ever smoker | 0.94(0.27-3.25) | 0.928 | 0.70(0.27-1.80) | 0.456 | 1.6(0.4-6.4) | 0.490 | 0.85(0.30-2.37) | 0.752 | 0.395 |
| Parous | 1.2(0.7-2.3) | 0.519 | 1.3(0.8-2.2) | 0.225 | 0.75(0.33-1.70) | 0.484 | 0.92(0.53-1.61) | 0.764 | 0.736 |
| History of GDM in previous pregnancy | 12.2(1.1-135.3) | 0.042 | 8.3(3.3-20.7) | <0.001 | 2.6(0.2-43.5) | 0.511 | 7.8(2.7-22.8) | <0.001 | <0.001 |
| Male fetus | 0.97(0.63-1.49) | 0.873 | 1.0(0.6-1.6) | 0.988 | 0.96(0.58-1.58) | 0.861 | 0.84(0.49-1.42) | 0.506 | 0.748 |

Adjusted for maternal age, pre-pregnancy BMI, GWG velocity in early pregnancy (z score), alcohol consumption, family history of diabetes, family history of hypertension, smoking status, parity, history of GDM in previous pregnancy, fetal sex.

For adjusted model, 74.4% of SBC subjects (546 out of 734) were used while 82.9% of GUSTO subjects (561 out of 677) were used.

*P value for the difference between the two cohorts in the adjusted model.

Supplementary Table 8. Effect of GWG velocity among women of different pre-pregnancy BMI and fetal sex on GDM development defined by IADPSG criteria.

| GDM | Shanghai Birth cohort  Unadjusted | | GUSTO Birth Cohort  Unadjusted | | Shanghai Birth cohort  Adjusted | | GUSTO Birth Cohort  Adjusted | | P-value* |
| --- | --- | --- | --- | --- | --- | --- | --- | --- | --- |
|  | OR (95% CI) | P-value | OR (95% CI) | P-value | OR (95% CI) | P-value | OR (95% CI) | P-value |  |
| Maternal age | 1.1(1.0-1.2) | 0.002 | 1.08(1.03-1.14) | 0.004 | 1.1(1.0-1.2) | 0.032 | 1.06(0.99-1.13) | 0.088 | 1.000 |
| GWG velocity in early pregnancy (z score) | 1.1(0.9-1.3) | 0.476 | 1.2(0.9-1.5) | 0.225 | 1.3(0.9-1.8) | 0.194 | 1.2(0.8-1.9) | 0.396 | 0.724 |
| Pre-pregnancy BMI |  |  |  |  |  |  |  |  |  |
| <18.5 | 0.82(0.40-1.67) | 0.579 | 0.58(0.22-1.53) | 0.274 | 1.1(0.5-2.4) | 0.896 | 0.69(0.26-1.88) | 0.472 | 0.524 |
| ≥18.5 to <23 | 1.0 |  | 1.0 |  | 1.0 |  | 1.0 |  |  |
| ≥23 to <27.5 | 2.5(1.5-4.0) | <0.001 | 1.5(0.8-2.8) | 0.190 | 2.5(1.4-4.4) | 0.002 | 1.3(0.7-2.6) | 0.422 | 0.007 |
| ≥27.5 | 3.7(1.7-8.0) | 0.001 | 4.3(2.0-9.1) | <0.001 | 3.7(1.4-9.9) | 0.009 | 3.8(1.7-8.7) | 0.002 | 0.878 |
| Alcohol consumption | 5.8(0.4-93.9) | 0.214 | 0.85(0.19-3.72) | 0.824 | 12.6(0.8-209.1) | 0.078 | 1.1(0.2-5.0) | 0.914 | <0.001 |
| Family history of diabetes | 2.3(1.3-4.3) | 0.008 | 1.3(0.7-2.2) | 0.388 | 1.8(0.9-3.7) | 0.077 | 0.68(0.35-1.33) | 0.262 | 0.024 |
| Family history of hypertension | 1.5(1.0-2.3) | 0.077 | 1.1(0.7-1.8) | 0.649 | 1.4(0.8-2.4) | 0.225 | 0.91(0.52-1.61) | 0.752 | 0.223 |
| Current or ever smoker | 0.94(0.27-3.25) | 0.928 | 0.70(0.27-1.80) | 0.456 | 1.9(0.4-7.8) | 0.391 | 0.87(0.31-2.44) | 0.791 | 0.264 |
| Parous | 1.2(0.7-2.3) | 0.519 | 1.3(0.8-2.2) | 0.225 | 0.69(0.30-1.61) | 0.389 | 0.94(0.53-1.66) | 0.827 | 0.630 |
| History of GDM in previous pregnancy | 12.2(1.1-135.3) | 0.042 | 8.3(3.3-20.7) | <0.001 | 2.5(0.1-45.5) | 0.541 | 8.1(2.7-23.8) | <0.001 | 0.001 |
| Male fetus | 0.97(0.63-1.49) | 0.873 | 1.0(0.6-1.6) | 0.988 | 0.98(0.58-1.63) | 0.922 | 0.84(0.49-1.45) | 0.526 | 0.714 |

Adjusted for maternal age, GWG velocity in early pregnancy, pre-pregnancy BMI group, alcohol consumption, family history of diabetes, family history of hypertension, smoking status, parity, history of GDM in previous pregnancy, fetal sex.

In the adjusted model, 74.4% of SBC subjects (546 out of 734) were used, 82.9% of GUSTO subjects (561 out of 677) were used.

*P value refers to the difference between cohorts in the adjusted model.

Interaction between GWG velocity and pre pregnancy BMI group and fetal sex were included in the model but not significant.

Overall interaction for GWG velocity and pre pregnancy BMI group: p=0.376 for SBC, p=0.726 for GUSTO

Interaction for GWG velocity and fetal sex: p=0.454 for SBC, p=0.811 for GUSTO

Supplementary Table 9. Effect of all risk factors among women of different citizenship status on GDM development defined by IADPSG criteria in GUSTO cohort.

| GDM | Unadjusted |  | Adjusted | |
| --- | --- | --- | --- | --- |
|  | OR (95% CI) | P-value | OR (95% CI) | P- value |
| Maternal age | 1.08(1.03-1.14) | 0.004 | 1.1(1.0 – 1.2) | 0.246 |
| Pre-pregnancy BMI | 1.13(1.06-1.21) | <0.001 | 1.1(0.9 – 1.2) | 0.470 |
| GWG at early pregnancy (z score) |  |  |  |  |
| <-1 | 1.0 |  | 1.0 |  |
| -1 to 1 | 1.2(0.5-2.8) | 0.662 | 1.0(0.4 – 3.0) | 0.935 |
| >1 | 1.4(0.5-3.8) | 0.529 | 0.77(0.19 – 3.15) | 0.714 |
| Alcohol consumption | 0.85(0.19-3.72) | 0.824 | 0.76(0.09 – 6.67) | 0.803 |
| Family history of diabetes | 1.3(0.7-2.2) | 0.388 | 0.68(0.25 – 1.85) | 0.455 |
| Family history of hypertension | 1.1(0.7-1.8) | 0.649 | 1.3(0.6 – 2.8) | 0.550 |
| Current or ever smoker | 0.70(0.27-1.80) | 0.456 | 2.0(0.5 – 7.7) | 0.323 |
| Parous | 1.3(0.8-2.2) | 0.225 | 1.1(0.5 – 2.4) | 0.775 |
| History of GDM in previous pregnancy | 8.3(3.3-20.7) | <0.001 | 26.4(2.6 – 269.8) | 0.006 |
| Male fetus | 1.0(0.6-1.6) | 0.988 | 0.80(0.37 – 1.70) | 0.553 |
| Citizenship status | 0.72(0.45 – 1.17) | 0.184 | 0.05(0 – 14.19) | 0.302 |

Adjusted for maternal age, pre-pregnancy BMI, GWG at early pregnancy (z score), alcohol consumption, family history of diabetes, family history of hypertension, smoking status, parity, history of GDM in previous pregnancy, fetal sex and citizenship status

In the adjusted model, 82.9% of GUSTO subjects (561 out of 677) were used.

Interaction between citizenship status and all risk factors were included in the model but not significant.

Overall interaction for citizenship status and GWG at early pregnancy: p=0.432.

Interaction for citizenship status and maternal age, pre-pregnancy BMI, alcohol consumption, family history of diabetes, family history of hypertension, smoking status, parity, history of GDM in previous pregnancy and fetal sex: p=0.845, p=0.151, p=0.746, p=0.992, p=0.235, p=0.114, p=0.414, p=0.296 and p=0.811, respectively.

Supplementary Table 10. Effect of weight gain among women of different pre-pregnancy BMI and citizenship status on GDM development defined by IADPSG criteria in GUSTO cohort.

| GDM | Unadjusted | | Adjusted | |
| --- | --- | --- | --- | --- |
|  | OR (95% CI) | P-value | OR (95% CI) | P- value |
| Maternal age | 1.08(1.03-1.14) | 0.004 | 1.1(1.0 – 1.2) | 0.198 |
| GWG at early pregnancy (z score) | 1.1(0.9-1.5) | 0.277 | 0.75(0.30 – 1.88) | 0.542 |
| Pre-pregnancy BMI |  |  |  |  |
| <18.5 | 1.0 |  | 1.0 |  |
| ≥18.5 to <23 | 1.7(0.7 – 4.5) | 0.274 | 1.1(0.3 – 4.2) | 0.859 |
| ≥23 to <27.5 | 2.6(0.9 – 7.4) | 0.073 | 0.44(0.07 – 2.71) | 0.376 |
| ≥27.5 | 7.4(2.4 – 22.8) | <0.001 | 11.6(1.3 – 102.4) | 0.027 |
| Alcohol consumption | 0.85(0.19-3.72) | 0.824 | 0.71(0.08 – 6.72) | 0.763 |
| Family history of diabetes | 1.3(0.7-2.2) | 0.388 | 0.41(0.12 – 1.33) | 0.137 |
| Family history of hypertension | 1.1(0.7-1.8) | 0.649 | 1.4(0.6 – 3.1) | 0.422 |
| Current or ever smoker | 0.70(0.27-1.80) | 0.456 | 2.1(0.5 – 8.2) | 0.309 |
| Parous | 1.3(0.8-2.2) | 0.225 | 1.1(0.5 – 2.4) | 0.858 |
| History of GDM in previous pregnancy | 8.3(3.3-20.7) | <0.001 | 37.1(3.3 – 416.5) | 0.003 |
| Male fetus | 1.0(0.6-1.6) | 0.988 | 0.87(0.39 – 1.91) | 0.721 |
| Citizenship status | 0.72(0.45 – 1.17) | 0.184 | 0.93(0.01 – 93.52) | 0.975 |

Adjusted for maternal age, pre-pregnancy BMI, GWG at early pregnancy (z score), alcohol consumption, family history of diabetes, family history of hypertension, smoking status, parity, history of GDM in previous pregnancy, fetal sex and citizenship status

In the adjusted model, 82.9% of GUSTO subjects (561 out of 677) were used.

Interaction between GWG and pre pregnancy BMI group and fetal sex, interaction between citizenship status and all risk factors were included in the model but not significant.

Overall interaction for GWG and pre pregnancy BMI group: p=0.876.

Interaction for GWG and fetal sex: p=0.640.

Overall interaction for citizenship status and pre pregnancy BMI group: p=0.050.

Interaction for citizenship status and maternal age, GWG, alcohol consumption, family history of diabetes, family history of hypertension, smoking status, parity, history of GDM in previous pregnancy and fetal sex: p=0.799, p=0.442, p=0.579, p=0.510, p=0.211, p=0.129, p=0.447, p=0.273 and p=0.928, respectively.

Supplementary Table 11. Effect of GWG velocity among women of different citizenship status on GDM development defined by IADPSG criteria in GUSTO cohort.

| GDM | Unadjusted |  | Adjusted | |
| --- | --- | --- | --- | --- |
|  | OR (95% CI) | P-value | OR (95% CI) | P- value |
| Maternal age | 1.08(1.03-1.14) | 0.004 | 1.1(1.0 – 1.2) | 0.267 |
| Pre-pregnancy BMI | 1.13(1.06-1.21) | <0.001 | 1.1(0.9 – 1.2) | 0.457 |
| GWG velocity in early pregnancy (z score) | 1.2(0.9 – 1.5) | 0.225 | 1.0(0.7 – 1.4) | 0.995 |
| Alcohol consumption | 0.85(0.19-3.72) | 0.824 | 0.79(0.09 – 6.90) | 0.828 |
| Family history of diabetes | 1.3(0.7-2.2) | 0.388 | 0.69(0.26 – 1.85) | 0.459 |
| Family history of hypertension | 1.1(0.7-1.8) | 0.649 | 1.3(0.6 – 2.8) | 0.556 |
| Current or ever smoker | 0.70(0.27-1.80) | 0.456 | 2.0(0.5 – 7.5) | 0.332 |
| Parous | 1.3(0.8-2.2) | 0.225 | 1.1(0.5 – 2.4) | 0.796 |
| History of GDM in previous pregnancy | 8.3(3.3-20.7) | <0.001 | 26.5(2.6 – 269.6) | 0.006 |
| Male fetus | 1.0(0.6-1.6) | 0.988 | 0.78(0.37 – 1.65) | 0.511 |
| Citizenship status | 0.72(0.45 – 1.17) | 0.184 | 0.11(0 – 19.9) | 0.402 |

Adjusted for maternal age, pre-pregnancy BMI, GWG velocity in early pregnancy (z score), alcohol consumption, family history of diabetes, family history of hypertension, smoking status, parity, history of GDM in previous pregnancy, fetal sex and citizenship status

In the adjusted model, 82.9% of GUSTO subjects (561 out of 677) were used.

Interaction between citizenship status and all risk factors were included in the model but not significant.

Interaction for citizenship status and maternal age, pre-pregnancy BMI, GWG velocity, alcohol consumption, family history of diabetes, family history of hypertension, smoking status, parity, history of GDM in previous pregnancy and fetal sex: p=0.891, p=0.173, p=0.218, p=0.723, p=0.953, p=0.263, p=0.118, p=0.467, p=0.302 and p=0.729, respectively.

Supplementary Table 12. Effect of weight gain among women of different pre-pregnancy BMI and citizenship status on GDM development defined by IADPSG criteria in GUSTO cohort.

| GDM | Unadjusted | | Adjusted | |
| --- | --- | --- | --- | --- |
|  | OR (95% CI) | P-value | OR (95% CI) | P- value |
| Maternal age | 1.08(1.03-1.14) | 0.004 | 1.1(1.0 – 1.2) | 0.187 |
| GWG velocity in early pregnancy (z score) | 1.2(0.9 – 1.5) | 0.225 | 0.91(0.38 – 2.15) | 0.826 |
| Pre-pregnancy BMI |  |  |  |  |
| <18.5 | 1.0 |  | 1.0 |  |
| ≥18.5 to <23 | 1.7(0.7 – 4.5) | 0.274 | 1.1(0.3 – 4.2) | 0.838 |
| ≥23 to <27.5 | 2.6(0.9 – 7.4) | 0.073 | 0.44(0.07 – 2.70) | 0.374 |
| ≥27.5 | 7.4(2.4 – 22.8) | <0.001 | 10.6(1.2 – 92.7) | 0.032 |
| Alcohol consumption | 0.85(0.19-3.72) | 0.824 | 0.73(0.08 – 6.80) | 0.779 |
| Family history of diabetes | 1.3(0.7-2.2) | 0.388 | 0.42(0.13 – 1.38) | 0.152 |
| Family history of hypertension | 1.1(0.7-1.8) | 0.649 | 1.4(0.6 – 3.2) | 0.424 |
| Current or ever smoker | 0.70(0.27-1.80) | 0.456 | 2.0(0.5 – 8.1) | 0.312 |
| Parous | 1.3(0.8-2.2) | 0.225 | 1.1(0.5 – 2.4) | 0.852 |
| History of GDM in previous pregnancy | 8.3(3.3-20.7) | <0.001 | 35.1(3.2 – 388.3) | 0.004 |
| Male fetus | 1.0(0.6-1.6) | 0.988 | 0.88(0.40 – 1.93) | 0.747 |
| Citizenship status | 0.72(0.45 – 1.17) | 0.184 | 0.92(0.01 – 92.46) | 0.973 |

Adjusted for maternal age, pre-pregnancy BMI, GWG velocity in early pregnancy (z score), alcohol consumption, family history of diabetes, family history of hypertension, smoking status, parity, history of GDM in previous pregnancy, fetal sex and citizenship status

In the adjusted model, 82.9% of GUSTO subjects (561 out of 677) were used.

Interaction between GWG velocity and pre pregnancy BMI group and fetal sex, interaction between Singapore citizenship status and all risk factors were included in the model but not significant.

Overall interaction for GWG velocity and pre pregnancy BMI group: p=0.832.

Interaction for GWG velocity and fetal sex: p=0.876.

Overall interaction for citizenship status and pre pregnancy BMI group: p=0.050.

Interaction for citizenship status and maternal age, GWG velocity, alcohol consumption, family history of diabetes, family history of hypertension, smoking status, parity, history of GDM in previous pregnancy and fetal sex: p=0.809, p=0.243, p=0.563, p=0.529, p=0.210, p=0.126, p=0.443, p=0.285 and p=0.901, respectively.

Supplementary Table 13. Comparison of child birth size, weight and preterm births using 1999 WHO criteria.

| Characteristics | SBC  (n=734) | | | GUSTO  (n=677) | | | p-value  (within mothers with GDM) |
| --- | --- | --- | --- | --- | --- | --- | --- |
|  | Without GDM | With GDM | p-value | Without GDM | With GDM | p-value |  |
| **Birthweight for gestational age** |  |  | 0.202 |  |  | 0.972 | 0.395 |
| Small | 62(10.5%) | 9(8.0%) |  | 49(9.9%) | 14(10.6%) |  |  |
| Large | 84(14.2%) | 23(20.5%) |  | 71(14.4%) | 19(14.4%) |  |  |
| **Preterm birth** | 27(4.4%) | 10(8.3%) | 0.077 | 30(6.1%) | 16(12.1%) | 0.018 | 0.313 |
| **Birthweight (g)** | 3391.8±430.7 | 3339.7±508.4 | 0.321 | 3112.7±436.7 | 3045.7±464.9 | 0.123 | <0.001 |

Missing values were excluded.

Supplementary Table 14. Comparison of child birth size, weight and preterm births using IADPSG criteria.

| Characteristics | SBC  (n=734) | | | GUSTO  (n=677) | | | p-value  (within mothers with GDM) |
| --- | --- | --- | --- | --- | --- | --- | --- |
|  | Without GDM | With GDM | p-value | Without GDM | With GDM | p-value |  |
| **Birthweight for gestational age** |  |  | 0.373 |  |  | 0.855 | 0.539 |
| Small | 63(10.4%) | 8(8.3%) |  | 54(9.8%) | 9(11.8%) |  |  |
| Large | 88(14.5%) | 19(19.8%) |  | 79(14.4%) | 11(14.5%) |  |  |
| **Preterm birth** | 29(4.6%) | 8(7.7%) | 0.184 | 38(6.9%) | 8(10.5%) | 0.257 | 0.509 |
| **Birthweight (g)** | 3391.5±435.2 | 3332.3±495.6 | 0.236 | 3105.5±438.7 | 3049±475.1 | 0.299 | <0.001 |

Missing values were excluded.
